# Relation of Cumulative Low-Level Lead Exposure to Depressive and Phobic Anxiety Symptom Scores in Middle-Age and Elderly Women

**DOI:** 10.1289/ehp.1104395

**Published:** 2012-02-29

**Authors:** Ki-Do Eum, Susan A. Korrick, Jennifer Weuve, Olivia Okereke, Laura D. Kubzansky, Howard Hu, Marc G. Weisskopf

**Affiliations:** 1 Department of Environmental Health, Harvard School of Public Health, Boston, Massachusetts, USA; 2 Channing Laboratory, Department of Medicine, Brigham and Women’s Hospital and Harvard Medical School, Boston, Massachusetts, USA; 3 Rush Institute for Healthy Aging, Rush University Medical Center, Chicago, Illinois, USA; 4 Department of Epidemiology, Harvard School of Public Health, Boston, Massachusetts, USA; 5 Department of Society, Human Development and Health, Harvard School of Public Health, Boston, Massachusetts, USA; 6 Department of Environmental Health Sciences, University of Michigan School of Public Health, Ann Arbor, Michigan, USA

**Keywords:** anxiety, depression, environmental exposure, epidemiology, lead, longitudinal study

## Abstract

Background: Different lines of evidence suggest that low-level lead exposure could be a modifiable risk factor for adverse psychological symptoms, but little work has explored this relation.

Objective: We assessed whether bone lead—a biomarker of cumulative lead exposure—is associated with depression and anxiety symptoms among middle-age and elderly women.

Methods: Participants were 617 Nurses’ Health Study participants with K-shell X-ray fluorescence bone lead measures and who had completed at last one Mental Health Index 5-item scale (MHI-5) and the phobic anxiety scale of the Crown-Crisp Index (CCI) assessment at mean ± SD age of 59 ± 9 years (range, 41–83 years). With exposure expressed as tertiles of bone lead, we analyzed MHI-5 scores as a continuous variable using linear regression and estimated the odds ratio (OR) of a CCI score ≥ 4 using generalized estimating equations.

Results: There were no significant associations between lead and either outcome in the full sample, but associations were found among premenopausal women and women who consistently took hormone replacement therapy (HRT) between menopause and bone lead measurement (*n* = 142). Compared with women in the lowest tertile of tibia lead, those in the highest scored 7.78 points worse [95% confidence interval (CI): –11.73, –3.83] on the MHI-5 (*p*-trend = 0.0001). The corresponding OR for CCI ≥ 4 was 2.79 (95% CI: 1.02, 7.59; *p*-trend = 0.05). No consistent associations were found with patella lead.

Conclusions: These results provide support for an association of low-level cumulative lead exposure with increased depressive and phobic anxiety symptoms among older women who are premenopausal or who consistently take postmenopausal HRT.

The public health burden of psychiatric disorders such as depression and anxiety is tremendous—an estimated 450 million people worldwide suffer from psychiatric disorders [World Health Organization (WHO) 2001]. In the United States, the lifetime prevalence of major depressive disorder [using survey ascertainment of symptoms consistent with *Diagnostic and Statistical Manual of Mental Disorders* (DSM) diagnostic criteria (American Psychiatric Association 1987, 1994)] is approximately 10% for men and 20% for women (Kessler et al. 1994; Steffens et al. 2000). Furthermore, very high percentages of older people suffer from depressive (10–30%) or anxiety (~ 20%) symptoms that do not meet full diagnostic criteria (Beekman et al. 1999; Himmelfarb and Murrell 1984) but are associated with excess morbidity and functional impairment (Beekman et al. 1999; de Beurs et al. 1999; Himmelfarb and Murrell 1984; Lyness et al. 2007), greater risk of subsequent clinical diagnoses of depression and anxiety (Horwath et al. 1992; van’t Veer-Tazelaar et al. 2009), and greater health care service use and costs (de Beurs et al. 1999; Simon et al. 1995). Improved understanding of risk factors for anxiety and depressive symptoms could help address the public health burden of these disorders.

Few studies have considered the possible link between environmental toxicant exposures at low levels found in the community and mental health outcomes, despite the evidence from occupational studies that aspects of mood may be particularly sensitive to such exposures (Johnson et al. 1987). Given that many toxicant exposures are potentially modifiable, this group of potential risk factors for mental health outcomes should be of interest. Lead exposure is of particular interest because it is an established neurotoxicant (Bressler et al. 1999) with known effects on several brain systems implicated in depression and anxiety, including monoaminergic signaling (Kala and Jadhav 1995; Lasley et al. 1984; Virgolini et al. 2005) and the hypothalamic–pituitary–adrenal (HPA) axis (Cory-Slechta et al. 2008; Virgolini et al. 2005). Lead exposure is also a known risk factor for many cardiovascular end points (Eum et al. 2011; Navas-Acien et al. 2007; Weisskopf et al. 2009), which have also been demonstrated to be associated with adverse psychological symptoms, particularly in older adults (Alexopoulos et al. 1997; Vink et al. 2008). Furthermore, several studies among occupationally exposed adults have found lead exposure to be related to mood disorders and psychological symptoms (Balbus-Kornfeld et al. 1995; Schwartz et al. 2005; Shih et al. 2007). However, large-scale epidemiological studies of low-level environmental exposure to lead and psychological symptoms have been conducted in only two settings, the Normative Aging Study (NAS) and the National Health and Nutrition Examination Survey (NHANES), with some studies (Bouchard et al. 2009; Rajan et al. 2007; Rhodes et al. 2003), but not all (Golub et al. 2010), suggesting associations.

Only one of the prior studies of low-level lead exposure and mental health included older women, and even in that study the average age was only 46.5 years (Golub et al. 2010). Focusing on women of all ages is critical because the prevalence of mood disorders and anxiety is twice as common in women as in men (Beekman et al. 1999; Kessler et al. 1994), and measures of cumulative exposure to lead may be of particular importance. Therefore, we explored the association between cumulative lead exposure—as measured by lead in bone—and mental health among middle-age and elderly women participating in the Nurses’ Health Study (NHS). Because of the influence of menopause and subsequent hormone replacement therapy (HRT) on bone dynamics (Nelson et al. 2002; Seeman 2003), including the accumulation of lead in bone, we also examined the influence of these factors.

## Materials and Methods

*Study population.* The NHS is a cohort of 121,700 registered nurses recruited between the ages of 30 and 55 years in 1976 and followed up via biennial mailed questionnaires (Colditz et al. 1997). Our study population was drawn from two subsamples of the NHS cohort that was evaluated for lead exposure. The first is a sample of women who participated in a case–control study of lead exposure and hypertension (Korrick et al. 1999). Women were invited to participate if they lived in the Greater Boston metropolitan area, Massachusetts, were not obese (body mass index < 29 kg/m^2^), and did not have a history of major, chronic disease—mental health problems were not an exclusion criterion. Women who first reported a diagnosis of hypertension between 1990 and 1994 were invited to participate as cases; women free of major chronic disease participted as controls and were frequency matched to cases by 5-year age groups. In total, between 1993 and 1995, 302 NHS participants agreed to participate.

The women in the second sample were recruited for a study of lead exposure and osteoporosis. Similar eligibility criteria used for controls in the hypertension study were applied, with participants being free of chronic diseases (not including mental health problems) up to the time of recruitment (2001 through 2004), at which time they underwent their lead exposure measurements. This sample comprised 320 NHS participants. Altogether, lead content was measured in cortical (tibia) and trabecular (patella) bone in 621 and 620 women, respectively, from these two studies. After providing a complete description of the study to the participants, written informed consent was obtained before participation in each substudy. The present study was approved by the institutional review boards of the Brigham and Women’s Hospital and the Harvard School of Public Health.

**Figure 1:**
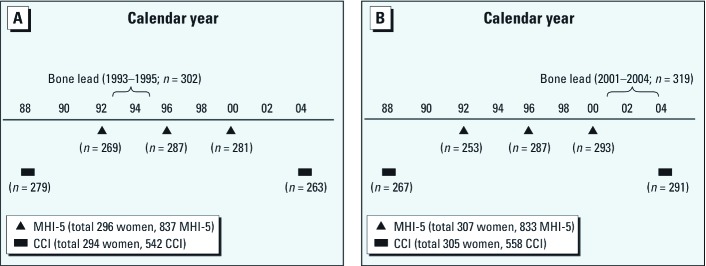
Timeline of MHI-5 and CCI assessments among women with bone lead measurements from study subsample 1 (*A*) and subsample 2 (*B*), as described in “Materials and Methods.”

Measures of psychological symptoms were obtained as part of several of the regular biennial NHS mailed questionnaires. Depressive symptoms were measured using the Mental Health Index 5-item (MHI-5) subscale, which was included on the 1992, 1996, and 2000 questionnaires. Anxiety symptoms were measured using the phobic anxiety scale of the Crown-Crisp Index (CCI) which was included on the 1988 and 2004 questionnaires. Of the 621 women who participated in the lead exposure studies, 617 completed at least one psychological symptom assessment. Of these 617 women, 613 (99.4%) completed at least one MHI-5 questionnaire, and 609 (98.7%) completed at least one CCI questionnaire. Individual women completed the MHI-5 in up to three separate study cycles for a total of 1,696 MHI-5 assessments, and the CCI in up to two separate study cycles for a total of 1,117 CCI assessments. We excluded 26 MHI-5 assessments (1.5%) and 17 CCI assessments (1.5%) for which data were missing on pack-years of smoking (*n* = 10 women). Thus, the final analytic sample included 603 women with 1,670 MHI-5 assessments (24 women with one, 91 with two, and 488 with three), and 599 women with 1,100 CCI assessments (98 women with one and 501 with two; Figure 1). All responses to the 2004 CCI (*n* = 554) occurred after bone lead measurement, whereas all responses to the 1988 CCI (*n* = 546) occurred before the bone lead measurements (mean ± SD, 10.0 ± 4.2 years; range, 4.4–15.9 years). Of the MHI-5 assessments, 1,102 (66.0%) occurred before the bone lead measurements (5.1 ± 3.5 years earlier; range, 0.4–12 years). Because bone lead reflects many years of past exposure, we included all psychological assessments in our primary analyses regardless of their timing with respect to bone lead measurement. Nonetheless, we also did sensitivity analyses restricted to those psychological assessments that occurred after bone lead measurement.

*Lead exposure assessment.* Participants visited the outpatient General Clinical Research Center of the Brigham and Women’s Hospital for measurement of lead content in their bone by K-shell X-ray fluorescence (KXRF) (Aro et al. 1994), expressed as micrograms of lead per gram of bone mineral (micrograms per gram). Bone lead measurements were taken at each woman’s midtibial shaft and patella. These sites are targets for bone lead research, because the half-life of lead in tibia and patella differs. In a cohort of older men, the half-life of lead in patella has been estimated to be on the order of years, whereas in tibia it is estimated to be on the order of decades (Wilker et al. 2011). Among women, however, faster bone turnover (Seeman 2003) likely makes these half-lives shorter.

When we began measuring the women’s bone lead, we used an instrument developed by ABIOMED Inc. (Danvers, MA) (Burger et al. 1990). In 1999, we replaced our prototype ABIOMED instrument with an upgraded instrument designed to improve measurement precision (Aro et al. 1994). Intercalibration data from persons who were measured on both instruments demonstrated a linear relationship between the two measurements with a slope of 0.87. Using this correction factor, we are able to combine data from our prototype and upgraded KXRF machines (Nie et al. 2008). To reduce the impact of any additional scaling differences in these readings on our epidemiologic analyses, we included in all of our bone lead regression models a term for lead substudy, which effectively adjusts for instrument, because women from the hypertension substudy were assessed on the ABIOMED instrument, and women from the osteoporosis substudy were assessed on the upgraded instrument.

*Psychological symptom assessment.* Symptoms of depression and anxiety were assessed with the MHI-5 and the CCI, respectively, each of which has been validated and has an extensive history in research and been used in populations of a similar age as ours (Albert et al. 2005; Beusterien et al. 1996; Burgess et al. 1987; Friedman et al. 2005). The MHI-5 is a five-item subscale derived from the Short Form-36 health status survey designed to capture, among other aspects of psychological functioning, psychological distress versus well-being (Ware and Sherbourne 1992). The MHI-5 asks respondents how much of the time over the past month (all, most, good bit, some, little, or none; ranked 1–6) they felt nervous, felt so down that nothing could cheer them up, felt calm and peaceful, felt down and blue, or felt happy. The two positively worded questions are reverse coded so that lower scores indicate more depressive symptoms; all item scores are summed, and then the sum is rescaled to obtain a total score ranging from 0 to 100 (Ware et al. 2000). An MHI-5 score < 60 denotes the presence of severe depressive symptoms and predicts major depression as identified using the Mini-International Neuropsychiatric Interview Major Depressive Episode module with high sensitivity and specificity among adults ≥ 65 years of age (Friedman et al. 2005). We also used an alternative cutoff score of < 53, which has been validated for identifying major depression in younger populations (Berwick et al. 1991; Holmes 1998).

The phobic anxiety scale of the CCI measures personality symptoms of phobic anxiety (Crown and Crisp 1966). It is a brief self-rating inventory of eight questions on common phobias—such as fear of enclosed spaces, illness, going out alone, heights, and crowds—with two yes or no questions (scored 2 or 0) and six three-level response questions (e.g., never/sometimes/often or not at all/moderately/very; scored 0/1/2). Scores range from 0 to 16, with higher scores corresponding with higher levels of phobic anxiety. The CCI has been validated in psychiatric outpatient clinic settings and found to discriminate patients with anxiety disorders and agoraphobia from those with other mental health disorders (Mavissakalian and Michelson 1981). The validity of the phobic anxiety subscale of the CCI in the NHS population has been tested previously, where scores were shown to be associated with use of tranquilizer medications (Albert et al. 2005). A CCI score of ≥ 4 has been related to several adverse outcomes in the NHS population, for example, Parkinson’s disease (Weisskopf et al. 2003), coronary heart disease, and sudden coronary death (Kawachi et al. 1994; Whang et al. 2009).

*Statistical analysis.* We used the generalized linear model framework to analyze repeated outcome measurements and used an unstructured covariance matrix to account for correlations in scores within individuals. Primary analyses treated MHI-5 scores as a continuous variable. Because the distribution of scores on the CCI was skewed, we used generalized estimating equations to estimate odds ratios (ORs) and 95% confidence intervals (CIs) for scoring ≥ 4 on any CCI measurement (i.e., high phobic anxiety; *n* = 188 women, 244 assessments). We conducted additional analyses using this same approach to estimate ORs for scoring < 60 on the MHI-5 (i.e., severe depressive symptoms; *n* = 96 women, 121 assessments) or the alternative cutoff of < 53. When using dichotomous outcomes, additional sensitivity analyses were performed that included as “cases” women taking antidepressant or antianxiety medication who did not meet criteria for major depression by MHI-5 scores (*n* = 63 women; 84 observations) or high phobic anxiety by CCI scores (*n* = 56 women; 56 observations)—along with women who were cases based on their symptom scores. For women missing one or two CCI responses (*n* = 55 women, 57 observations) or one MHI-5 response (*n* = 44 women, 46 observations), we used the standard approach of imputing the total score for the scale by dividing their score by the fraction of questions answered and rounding to the nearest integer (DeVellis 1991; Ware et al. 2000).

We specifically addressed the potential impact of menopause status in separate analyses. Menopause is associated with increased bone turnover that mobilizes stored lead from bone into circulating blood, increasing blood lead levels (Korrick et al. 2002). An increased rate of bone remodeling means that the lead concentration in bone is increasingly in flux during this period, which may compromise the utility of the bone lead concentration as a measure of cumulative lead exposure. Bone turnover with menopause is reduced in women who have taken HRT (Nelson et al. 2002). Therefore, we conducted additional analyses restricted to women either who were premenopausal at bone lead measurement (*n* = 45) or who had consistently taken HRT between menopause and bone lead measurement (*n* = 97). Menopausal status was based on each woman’s self report on the regular NHS biennial questionnaires of permanent cessation of natural menses, as well as the age at which this occurred. For women who underwent hysterectomy without bilateral oophorectomy, a life table was used to assign an age at menopause based on the date of surgery, smoking, and hormonal status.

We performed separate analyses for patella and tibia bone lead biomarkers. Bone lead tertiles were calculated based on the distributions among women who were premenopausal or consistently on HRT between menopause and bone lead measurements. Covariates in our models were obtained from biennial questionnaires and included age (years) at psychological symptom measurement, education (registered nurse, bachelor’s degree, master’s or doctorate degree), alcohol consumption (tertiles of grams per day: < 1.8, 1.8–8.69, ≥ 8.7), and pack-years of smoking (0 and tertiles among smokers: 1–8.9, 9–21.9, ≥ 22), because these variables have been associated with lead exposure and psychological symptoms (Bjelland et al. 2008; Boden and Fergusson 2011; Hu et al. 1996; Kessler et al. 1994; Korrick et al. 2002; Theppeang et al. 2008; Wiesbeck et al. 2008). We also included husband’s education (high school or less, college education, graduate school, or not married) and employment status at psychological symptom measurement (retired/homemaker, part-time worker, full-time worker) as indicators of socioeconomic status, which may be related to both lead exposure and psychological symptoms (Kessler et al. 1994; Theppeang et al. 2008). We adjusted for substudy to account for both the different KXRF machines used in the two substudies, as well as secular trends in lead exposure and possibly psychological symptom reporting, and age (years) at bone lead measurement, which is related to measured bone lead concentrations (Wilker et al. 2011). About half of the participants were women from a case–control study of hypertension. Because the selection of hypertension cases and controls could have introduced selection bias, we conducted additional sensitivity analyses adjusting for hypertensive status in this substudy. Hypertension cases were identified based on self-reported physician diagnosis on the NHS biennial questionnaires. We also conducted sensitivity analyses adding adjustment for whether the nurse’s parents owned their own home when she was born, an additional socioeconomic status indicator that reflects status in childhood. The women responded to items on some covariate data in more than one questionnaire cycle, and for our analyses, we used responses from the NHS questionnaire closest to the psychological symptom assessment. We used missing indicator variables for the small amount of missing data. Tests for trend with increasing lead concentration were computed by including a continuous term for lead that was formed by assigning to each woman the median lead concentration of the tertile in which she was classified. This approach minimizes the influence of extreme exposure values. A two-sided *p*-value ≤ 0.05 was considered statistically significant. We conducted all analyses in SAS (version 9; SAS Institute Inc., Cary, NC).

## Results

The mean ± SD (range) age of women in our study was 60.9 ± 6.0 (46–74) years at the time of lead measurement. Age at the time of MHI-5 assessment was 59.4 ± 7.3 (45–79) years, and at CCI assessment was 59.2 ± 10.2 (41–83) years. The mean ± SD levels of tibia lead and patella lead were 10.3 ± 9.5 μg/g and 12.5 ± 11.2 μg/g, respectively. As has been previously reported (Korrick et al. 2002), both patella and tibia bone lead levels were higher with older age, more pack-years of smoking, and alcohol consumption (Table 1). There were less consistent associations between other covariates and bone lead levels. Although MHI-5 and CCI were designed to target different types of psychological distress, they are often correlated because depression and anxiety are highly comorbid (Byers et al. 2010; Kessler et al. 1994). In our population, MHI-5 and CCI assessments were done in different years of the NHS, and Spearman correlations for these two exams, which ranged from –0.22 to –0.34, were modest.

**Table 1:** Levels of lead exposure biomarkers (μg/g) by characteristic among women with measures of psychological symptoms (n = 617).

Tibia lead					Patella lead				
Characteristica	n	Mean ± SD	Median	nb	Mean ± SD	Median
Age at lead exposure assessment (years)												
< 55		95		9.2 ± 7.4		8.7		95		12.8 ± 9.6		11.3
55–59		143		9.2 ± 8.6		9.0		143		10.8 ± 10.0		10.4
60–64		194		9.3 ± 9.7		9.0		194		12.1 ± 11.1		13.0
65–69		142		12.9 ± 10.3		13.0		142		13.1 ± 12.4		12.1
≥ 70		43		12.9 ± 11.4		12.2		42		17.3 ± 13.6		16.1
Education in 1992												
Registered nurse diploma		351		10.4 ± 9.6		10.0		350		13.3 ± 10.7		12.2
Bachelor’s degree		161		10.6 ± 8.8		9.6		161		10.6 ± 12.1		11.3
Master’s or doctorate degree		72		8.6 ± 10.9		8.0		72		12.6 ± 12.1		11.7
Missing		33		12.2 ± 9.6		11.0		33		12.2 ± 10.0		14.0
Husband education in 1992												
High school or less		144		11.6 ± 10.1		10.7		143		14.2 ± 12.6		13.0
College education		157		10.0 ± 9.3		9.6		157		12.1 ± 11.7		11.3
Graduate school		168		9.5 ± 10.0		9.0		168		11.8 ± 10.4		11.7
No husband (not married)		70		10.6 ± 8.8		10.0		70		12.2 ± 11.0		12.2
Missing		78		10.4 ± 8.4		9.3		78		11.7 ± 9.3		12.2
Employment status in 2004												
Retired/homemaker		305		11.5 ± 10.3		10.4		304		14.0 ± 11.1		13.0
Part-time worker		90		9.5 ± 9.1		10.0		90		9.0 ± 11.9		9.8
Full-time worker		170		8.9 ± 8.4		8.0		170		11.7 ± 11.1		12.2
Missing		52		9.6 ± 8.2		9.3		52		12.1 ± 9.8		12.2
Pack-years of cigarette smoking as of 2004												
0		239		9.6 ± 8.8		9.0		238		11.0 ± 10.4		11.7
1–8.9		110		9.2 ± 8.8		9.0		110		11.8 ± 8.8		11.3
9–21.9		122		10.8 ± 10.6		10.7		122		13.0 ± 13.0		10.7
22–80		136		12.1 ± 10.0		10.9		136		15.1 ± 12.2		14.0
Missing		10		11.8 ± 11.1		11.7		10		12.8 ± 13.0		11.3
Alcohol consumption in 2002 (g/day)												
< 1.8		218		9.3 ± 8.5		9.6		217		11.5 ± 10.5		12.0
1.8–8.69		189		10.4 ± 9.3		9.0		189		12.6 ± 11.7		12.2
≥ 8.7		210		11.3 ± 10.6		10.4		210		13.3 ± 11.5		12.2
Study subsample												
Lead and hypertension sample		302		11.7 ± 7.8		10.4		302		15.1 ± 9.5		13.5
Lead and osteoporosis sample		315		9.1 ± 10.8		8.0		314		9.9 ± 12.2		10.0
Postmenopausal hormone use at lead exposure assessment												
Premenopausal (never user)		45		9.1 ± 7.6		8.7		45		11.9 ± 10.4		12.2
Continuous user after menopause		97		9.6 ± 7.8		9.0		97		12.5 ± 9.1		11.3
Never user or inconsistent user		475		10.6 ± 10.0		10.0		474		12.5 ± 11.7		12.2
aVariables were determined in different years based on date of questionnaire or measure as part of the parent NHS. Bone lead levels were standardized to account for differences between measures done before versus after 1999. bn = 616 for patella lead analyses because of missing patella lead data.												

In analyses of all women, depressive symptoms were inconsistently worse with higher tibia lead levels (Table 2). Compared with the lowest tertile of tibia lead concentration, women in the middle tertile scored 1.70 MHI-5 points worse (95% CI: –3.75, 0.34), and those in the highest tertile scored 1.1 points worse (95% CI: –3.1, 0.94). At the time of bone lead measurement, most of the women (*n* = 572) were postmenopausal, with a mean ± SD of 12.2 ± 6.5 years since menopause. In analyses restricted to premenopausal women and postmenopausal women who were consistently on HRT, MHI-5 scores decreased monotonically with increasing tibia lead. Compared with women in the lowest tertile of tibia lead concentration, women in the middle and highest tertiles scored 4.31 (95% CI: –7.88, –0.74) and 7.78 (95% CI: –11.73, –3.83) points lower, respectively, on the MHI-5 (Table 2). In sensitivity analyses either restricting the analyses to MHI-5 assessments that occurred after bone lead measurement or restricting to women who scored < 4 on both CCI assessments, results were similar [see Supplemental Material, Table 1 (http://dx.doi.org/10.1289/ehp.1104395)].

**Table 2:** Adjusted^a^ differences in MHI-5 score by bone lead tertile.

All women					Premenopausal or postmenopausal on HRTb				
n (participants/MHI-5 assessments)	MHI-5			n (participants/MHI-5 assessments)	MHI-5		
	Lead biomarker	Score (mean ± SD)	Point difference (95% CI)		Score (mean ± SD)	Point difference (95% CI)
Tibia lead tertile (μg/g)												
< 7.0		202/558		81 ± 12		Reference		46/129		83 ± 10		Reference
7.0–11.5		159/440		79 ± 13		–1.70 (–3.75, 0.34)		48/134		78 ± 15		–4.31 (–7.88, –0.74)
> 11.5		242/672		80 ± 13		–1.06 (–3.05, 0.94)		47/126		76 ± 13		–7.78 (–11.73, –3.83)
p-Trend						0.33						0.0001
Total		603/1,670		80 ± 13				141/389		79 ± 13		
Patella lead tertile (μg/g)												
< 8.5		193/532		79 ± 12		Reference		47/128		80 ± 13		Reference
8.5–14.5		179/501		80 ± 13		1.02 (–1.06, 3.11)		49/135		78 ± 14		–0.66 (–5.00, 3.67)
> 14.5		230/634		80 ± 13		0.61 (–1.55, 2.78)		45/126		79 ± 12		0.51 (–3.91, 4.94)
p-Trend						0.64						0.77
Total		602/1,667		80 ± 13				141/389		79 ± 13		
aAdjusted for substudy group, age at bone lead and at MHI-5 measurement, education, husband’s education, alcohol consumption, pack-years of smoking, and employment status at MHI-5 measurement; lower scores indicate more depressive symptoms (lower MHI-5 scores indicate worse symptoms). bWomen who were either premenopausal at the time of bone lead measurement (n = 45) or consistently on HRT between menopause and bone lead measurement (n = 97).												

When lead was modeled as a continuous variable, there was a 5.63-point reduction (95% CI: –8.49, –2.78) in MHI-5 score per 1 SD higher tibia lead (9.5 μg/g) among premenopausal women and postmenopausal women consistently on HRT [see Supplemental Material, Table 1 (http://dx.doi.org/10.1289/ehp.1104395)]. When we dichotomized MHI-5 scores as < 60 (severe depression symptoms) or at least 60, among premenopausal women and postmenopausal women consistently on HRT (*n* = 37 MHI-5 assessments < 60), the OR for severe depression symptoms for women in the highest tertile of tibia lead was 3.71 (95% CI: 1.18, 11.64; *p*-trend = 0.02) compared with women in the lowest tertile. In analyses of all women, the OR for depression for women in the highest, compared with lowest, tibia tertile was 1.42 (95% CI: 0.81, 2.46). These results were similar using the alternative MHI-5 cutoff of < 53 (Berwick et al. 1991; Holmes 1998), but fewer women met this threshold, so confidence limits were much larger (data not shown).

A similar pattern was apparent for anxiety symptoms. Tibia bone lead concentration was not associated with CCI scores in the overall population (Table 3). The strongest association appeared in analyses among premenopausal and postmenopausal women consistently on HRT. In this subset, the ORs for severe phobic anxiety (CCI ≥ 4) for women in the middle and highest tertiles of tibia lead concentration, compared with those in the lowest tertile, were 1.52 (95% CI: 0.60, 3.82) and 2.79 (95% CI: 1.02, 7.59; *p*-trend = 0.05), respectively (Table 3). All of these results were similar in sensitivity analyses restricted to the CCI assessments that occurred after bone lead measurement [see Supplemental Material, Table 2 (http://dx.doi.org/10.1289/ehp.1104395)]. When lead was modeled as a continuous variable, the OR for high phobic anxiety was 2.06 (95% CI: 1.01, 4.22) per 1 SD higher tibia lead (9.5 μg/g) among premenopausal women and postmenopausal women consistently on HRT (see Supplemental Material, Table 2).

**Table 3:** Adjusted^a^ ORs and 95% CI of high phobic anxiety (CCI ≥ 4) by bone lead tertile.

All women (n = 599; 1,100 assessmentsb)			Premenopausal or postmenopausal on HRTc (n = 140; 261 assessments)		
Lead biomarkers	CCI ≥ 4 (yes/no)	OR (95% CI)	CCI ≥ 4 (yes/no)	OR (95% CI)
Tibia lead tertile (μg/g)								
< 7.0		78/288		Reference		11/73		Reference
7.0–11.5		66/265		0.84 (0.54, 1.32)		21/83		1.52 (0.60, 3.82)
> 11.5		100/303		1.10 (0.73, 1.64)		20/53		2.79 (1.02, 7.59)
p-Trend				0.62				0.05
Patella lead tertile (μg/g)								
< 8.5		80/270		Reference		16/71		Reference
8.5–14.5		78/254		0.96 (0.63, 1.47)		26/64		1.16 (0.41, 3.28)
> 14.5		86/330		0.75 (0.49, 1.16)		10/74		0.23 (0.07, 0.69)
p-Trend				0.17				0.003
aAdjusted for substudy group, age at bone lead and at CCI measurement, education, husband’s education, alcohol consumption, pack-years of smoking, and employment status at CCI measurement. bOnly 1,098 for patella lead analyses because of missing patella lead data. cWomen who were either premenopausal at the time of bone lead measurement (n = 45) or consistently on HRT between menopause and bone lead measurement (n = 97).								

In general, we found much weaker, inconsistent, or null associations between patella lead concentration and either outcome. However, among premenopausal women and postmenopausal women consistently on HRT, in MHI-5 analyses restricted to those assessments that occurred after bone lead measurement, there was some suggestion of higher MHI-5 score (less depression) with higher patella lead [see Supplemental Material, Table 1 (http://dx.doi.org/10.1289/ehp.1104395)]. There was also a significant decrease in anxiety symptoms in the highest tertile of patella bone lead among premenopausal women and postmenopausal women consistently on HRT (Table 3). However, this association was seen only in the highest tertile, and precision was poor because of small numbers (*n* = 10 “cases”); the results in this tertile likely also drove the significant association with the continuous patella lead term (see Supplemental Material, Table 2).

All results were essentially unchanged upon additional adjustment for hypertension at bone lead measurement or for whether the nurse’s parents owned their own home when she was born, an indicator of childhood socioeconomic status (data not shown). Analyses restricted to women with no missing MHI-5 or CCI responses showed similar results. Although there were too few premenopausal women to analyze this group separately, analyses restricted to postmenopausal women consistently on HRT between menopause and bone lead measurement (*n* = 97) were similar to analyses that also included women who were premenopausal (data not shown). Among women who were premenopausal and postmenopausal women who were consistently on HRT, analyses of dichotomized outcomes also yielded similar results when we included as “cases” women taking antidepressant or antianxiety medication—regardless of their MHI-5 or CCI score—along with women who were cases based on their symptom scores (data not shown).

## Discussion

We found increased depressive symptom scores and high phobic anxiety scores in association with increasing tibia, but not patella, bone lead concentration among women who were either premenopausal or postmenopausal and consistently on HRT between menopause and bone lead measurement. These associations were independent of age, education, husband’s education, cigarette smoking, alcohol intake, and employment status. These results were observed among women with low blood lead concentrations consistent with nonoccupational background population exposures and presumed relatively low cumulative exposures (Table 1). The geometric mean blood lead concentration among our women was 2.37 μg/dL, which is similar to levels seen among women of comparable age in NHANES data from the same time periods (Jackson et al. 2010; Pirkle et al. 1998).

That the association was seen among premenopausal and postmenopausal women on HRT, rather than among the full sample, could be the result of less bone loss in these women compared with postmenopausal women who did not consistently take HRT (Nelson et al. 2002). Specifically, associations may have been attenuated for all women combined if tibia bone lead levels were a less accurate marker of cumulative lead exposure in postmenopausal women that were not taking HRT, a possible result of measurement error resulting from variation in the degree of bone turnover among these women. A biological interaction between HRT and lead that results in greater effects among HRT users is an alternative possibility. That the results are due to chance in this subgroup also cannot be ruled out. We observed an unexpected inverse association between patella lead and anxiety symptoms among women who were premenopausal or postmenopausal on HRT, although this was likely driven by a small number of women with high patella lead concentrations [see Table 3; see also Supplemental Material, Table 2 (http://dx.doi.org/10.1289/ehp.1104395)]. There was also some suggestion of less MHI-5 depressive symptoms with higher patella lead in one of the subanalyses (see Supplemental Material, Table 1), although this did not reach statistical significance. Although we cannot dismiss the possibility that these associations are causal, they may also have arisen by chance. Otherwise, patella lead measures were generally not associated with MHI-5 or CCI reported symptoms (Tables 2 and 3). The lack of consistent findings with patella lead suggests that the relevant lead exposure window for depression or anxiety symptoms may be more long term and therefore better reflected by tibia lead, which has a longer half-life than patella lead. On the other hand, greater variability of patella lead measurements could also contribute to the predominantly null patella findings.

Limitations of this study include the possibility that our findings are driven by reverse causality. For example, given that phobic anxiety generally has onset at young ages (Kessler et al. 2005), early-life anxiety could affect socioeconomic position, smoking habits, or other risk factors for lead exposure and thereby increase later bone lead levels. Furthermore, it is possible that lower socioeconomic status earlier in life could independently predict both later lead exposure and later phobic anxiety (or possibly depression), thereby leading to a spurious association. However, both of these possibilities are argued against by the fact that our results were essentially unchanged when adjusted for a marker of childhood socioeconomic status and pack-years of smoking. All of this suggests that some phobic anxiety may be sensitive to later-life risk factors. Also, the association between lead and CCI phobic anxiety score may not be restricted to phobic anxiety because the symptoms captured by the CCI scale have some overlap with other types of anxiety (Burgess et al. 1987; Crown and Crisp 1966).

Our data are limited to the available MHI-5 and CCI assessments for our study population; thus, we cannot determine whether the observed phobic anxiety symptoms are an exacerbation of previous symptoms from a younger age. Furthermore, neither the MHI-5 nor the CCI are diagnostic tools. Despite these limitations, both assessments have demonstrated strong relations with clinical disorders. In comparing scores on the MHI-5 with DSM-based diagnoses, the area under the receiver operating characteristic curve was found to be quite high for depression (0.892) and anxiety disorders (0.739) (Berwick et al. 1991). The latter likely reflects both the fact that the MHI-5 is not completely specific to depression symptoms and that there is frequently comorbidity between depression and anxiety. The CCI has been found to discriminate well between patients with anxiety disorders and agoraphobia and patients with other mental health disorders (Mavissakalian and Michelson 1981). Furthermore, symptoms ascertained by these measures have been associated with a number of clinical outcomes known to correlate with clinical depression and/or anxiety such as Parkinson’s disease (Weisskopf et al. 2003), coronary heart disease, and sudden coronary death (Kawachi et al. 1994; Whang et al. 2009). Thus, despite limitations, there is strong evidence that the study measures capture clinically relevant symptoms.

Low-level environmental exposure to lead and psychological distress has been explored only in two other settings, older men in the NAS and adult men and women in NHANES. Among 526 NAS men (67 ± 7 years, mean ± SD), higher patella bone lead concentration was significantly associated with scoring worse on the phobic anxiety scale of the Brief Symptom Inventory (BSI), and higher blood, patella, and tibia lead were all associated with scoring worse on an index that combined the BSI anxiety, depression, and phobic anxiety scales (Rhodes et al. 2003). Slightly weaker associations were seen in a follow-up study of this cohort (Rajan et al. 2007). In data from 1,987 men and women 20–39 years of age in the 1999–2004 NHANES cycles, higher blood lead level was significantly associated with prevalent diagnoses of major depressive disorder and panic disorder based on the fourth edition of the DSM, but not generalized anxiety disorder as assessed with the World Health Organization Composite International Diagnostic Interview (Bouchard et al. 2009). Bone lead concentration was not available in these data. A later study of 4,159 adults ≥ 20 years of age in the 2005–2006 NHANES cycle did not find a significant association between blood lead and depression symptom scores on the Patient Health Questionnaire-9 (PHQ-9), although PHQ-9 scores increased with increasing quintiles of blood lead concentration (Golub et al. 2010).

Lead is a neurotoxicant (Bressler et al. 1999) with known effects on neural systems that underlie mood and anxiety symptoms. For example, lead exposure has been found to specifically disrupt signaling in monoaminergic signaling systems that are frequent targets for medications to treat these symptoms (Haider et al. 2005; Kala and Jadhav 1995; Lasley et al. 1984; Virgolini et al. 2005). Lead can alter synaptic signaling and second-messenger systems much more generally, to a large extent as a result of its ability to substitute for calcium (Garza et al. 2006), and thus disrupt multiple neurotransmitter systems that are implicated in mood disorders (Werner and Covenas 2010; Wu et al. 2008). Lead exposure also disrupts the HPA axis leading to altered glucocorticoid and catecholaminergic signaling (Cory-Slechta et al. 2008; Virgolini et al. 2005), with mood disorder as a proposed consequence (Cory-Slechta et al. 2008). HPA axis function, corticotrophin-releasing hormone, and cortisol levels are found to be altered among people with depression compared with population norms or controls without psychiatric disease (Holsboer 2000; Vreeburg et al. 2009). Lead also has well-known adverse effects on the cardiovascular system (Eum et al. 2011; Navas-Acien et al. 2007; Weisskopf et al. 2009), and many cardiovascular risk factors have been found to predict depression. There is a high rate of depression among people with hypertension, diabetes, coronary artery disease, and stroke, as well as frequent occurrence of white matter hyperintensities and silent stroke (on brain magnetic resonance imaging) in geriatric depression. This has led to a vascular theory of depression that posits these factors disrupt neural systems in the prefrontal region of the brain (Alexopoulos et al. 1997; Vink et al. 2008). Thus, lead exposure could be related to psychological distress in older women via cardiovascular effects.

Strengths of our study include having a large group of women with bone lead measurements—a marker of cumulative lead exposure—and extensive covariate data. One limitation is that the women who were recruited for the original lead exposure studies were, excepting the subset with hypertension, selected to be particularly healthy, which may have made it more difficult to identify associations between lead and psychological symptoms. Given lead’s association with a number of chronic diseases, including cardiovascular, renal, and neurocognitive disorders (Ekong et al. 2006; Navas-Acien et al. 2007; Shih et al. 2007), women excluded because of chronic disease may have been more likely to both have higher lead exposure and lead-related disorders, including psychiatric symptoms. We did not have data on family history of psychiatric disorders and so could not adjust analyses for this. An additional limitation is that we did not have standardized clinical assessments for psychiatric disorders. Although such assessments would have been useful for purposes of establishing DSM-criteria disorders, our questionnaires include reliable and valid measures that have been used extensively for research purposes (Albert et al. 2005; Berwick et al. 1991; Burgess et al. 1987; Kawachi et al. 1994; Mavissakalian and Michelson 1981; Weisskopf et al. 2003; Whang et al. 2009). Furthermore, there is growing consideration of the value of dimensional measurement of psychological symptoms, for example, characterizing symptoms continuously across a spectrum of severity as we have done (National Institute of Mental Health 2010). Nevertheless, we also found similar results using the cutoffs of < 60 or < 53 for the MHI-5, both of which have been validated against clinical measures (Berwick et al. 1991; Friedman et al. 2005; Holmes 1998).

## Conclusion

Our results provide support for associations between lead exposure and both depression and anxiety among older women with community-level exposures. Specifically, these findings were present among premenopausal women and postmenopausal women consistently on HRT. This may relate to the reduced effectiveness of bone lead as a cumulative marker of lead exposure in postmenopausal women not taking HRT, but this warrants further confirmation. These results were found among women with low lead exposure levels that were similar to their same-age peers in NHANES data. Thus, these findings underscore the need to consider environmental contaminants as risk factors for psychological distress.

## Supplemental Material

(139 KB) PDF
