# Supplementary material for: Relation of Cumulative Low-Level Lead Exposure to Depressive and Phobic Anxiety Symptom Scores in Middle-Age and Elderly Women
Source: Environ Health Perspect. 2012 Feb 29;120(6):817–23. doi: 10.1289/ehp.1104395 (PMC3385437; doi:10.1289/ehp.1104395)
Supplement: (139 KB) PDF [file ehp.1104395.s001.pdf]

Supplemental material

Relation of Cumulative Low-Level Lead Exposure to Depressive and Phobic Anxiety Symptom Scores in Middle-Aged and Elderly Women

Ki-Do Eum, Susan A. Korrick, Jennifer Weuve, Olivia Okereke, Laura D. Kubzansky, Howard Hu, Marc G. Weisskopf

Supplemental Material, Table 1. Adjusted[a] difference (beta estimates) and 95% confidence interval (CI) in MHI-5 score by bone lead concentration.

Lead Biomarkers	All women			Premenopaual or Postmenopausal on HRT[b]		
	N[c]/Obs[d]	Mean±SD MHI-5	MHI-5 points (95% CI)	N[c]/Obs[d]	Mean±SD MHI-5	MHI-5 points (95% CI)
Continuous tibia lead, per 9.5 µg/g (sd)	603/1,670	80±13	-0.72 (-2.17, 0.73)	141/389	79±13	-5.63 (-8.49, -2.78)
Tibia lead tertile (µg/g), prospective[e]						
<7.0	78/152	82±11	Ref	27/52	84±9	Ref
7.0-11.5	86/165	81±11	-0.56 (-3.41, 2.29)	27/52	79±12	-3.01 (-7.98, 1.97)
>11.5	130/251	81±13	-1.07 (-4.04, 1.90)	32/61	79±13	-5.40 (-10.95, 0.14)
p-trend			*0.48*			*0.06*
Total	294/568	81±12		86/165	81±12	
Tibia lead tertile (µg/g), CCI<4[f]						
<7.0	135/381	82±11	Ref	34/96	82±10	Ref
7.0-11.5	116/337	80±13	-2.14 (-4.44, 0.15)	35/100	80±14	-3.53 (-7.56, 0.50)
>11.5	167/482	81±12	-1.08 (-3.34, 1.18)	31/89	78±13	-7.22 (-11.62, -2.81)
p-trend			*0.40*			*0.001*
Total	418/1,200	81±12		100/285	80±12	
Continuous patella lead, per 11.2 µg/g (sd)	602/1,667	80±13	0.39 (-1.23, 2.01)	141/389	79±13	0.48 (-2.81, 3.77)
Patella lead tertile (µg/g), prospective[e]						
<8.5	54/106	80±12	Ref	22/43	78±12	Ref
8.5-14.5	106/206	81±12	1.38 (-2.14, 4.90)	33/63	81±12	5.08 (-0.61, 10.77)
>14.5	134/256	81±12	1.24 (-2.07, 4.55)	31/59	81±11	5.41 (-0.03, 10.86)
p-trend			*0.55*			*0.08*
Total	294/568	81±12		86/165	81±12	
Patella lead tertile (µg/g), CCI<4[f]						
<8.5	129/370	80±12	Ref	34/95	80±12	Ref
8.5-14.5	128/368	82±11	1.53 (-0.73, 3.78)	31/89	81±13	-0.52 (-5.09, 4.05)
>14.5	160/459	81±13	0.07 (-2.42, 2.55)	35/101	80±12	-1.12 (-5.94, 3.69)
p-trend			*0.91*			*0.65*
Total	417/1,197	81±12		100/285	80±12	

[a] Adjusted for substudy group, age at bone lead and at MHI-5 measurement, education, husband's education, alcohol consumption, pack-years of smoking, and employment status at MHI-5 measurement; lower scores indicate more depressive symptoms.
[b] Women who were either premenopausal at the time of bone lead measurement (n=45) or consistently on HRT between menopause and bone lead measurement (n=97). [c] Participants. [d] Individual MHI assessments. [e] Only MHI assessments after bone lead measurement.
[f] Only women who scored <4 on both CCI.

Supplemental Material, Table 2. Adjusted[a] odds ratios (OR) and 95% confidence interval (CI) of high phobic anxiety (CCI≥4) by bone lead concentration.

Lead Biomarkers	All women (N=599; 1,100 assessments[b])		Premenopausal or Postmenopausal on HRT[c] (N=140; 261 assessments)	
	CCI≥4[b] Yes/No	OR (95% CI)	CCI≥4[b] Yes/No	OR (95% CI)
Continuous tibia lead, per 9.5 µg/g (sd)	244/856	1.08 (0.81, 1.44)	52/209	2.06 (1.01, 4.22)
Tibia lead tertile (µg/g), prospective[d]				
<7.0	27/159	Ref	3/39	Ref
7.0-11.5	28/137	1.18 (0.65, 2.17)	7/45	2.63 (0.62, 11.13)
>11.5	47/156	1.61 (0.92, 2.81)	10/28	10.21 (1.60, 65.05)
p-trend		*0.09*		*0.02*
Continuous patella lead, per 11.2 µg/g (sd)	244/854	0.80 (0.59, 1.10)	52/209	0.33 (0.16, 0.69)
Patella lead tertile (µg/g), prospective[d]				
<8.5	30/148	Ref	6/37	Ref
8.5-14.5	34/132	1.32 (0.73, 2.37)	8/37	0.85 (0.12, 6.01)
>14.5	38/171	0.96 (0.53, 1.75)	6/38	0.32 (0.04, 2.24)
p-trend		*0.75*		*0.17*

[a] Adjusted for substudy group, age at bone lead and at CCI measurement, education, husband's education, alcohol consumption, pack-years of smoking, and employment status at CCI measurement.

[b] Only 1,098 for patella lead analyses because of missing patella lead data.

[c] Women who were either premenopausal at the time of bone lead measurement (n=45) or consistently on HRT between menopause and bone lead measurement (n=97).

[d] Only CCI assessments after bone lead measurement.
